# Genetic variability in *IGF-1* and *IGFBP-3* and body size in early life

**DOI:** 10.1186/1471-2458-12-659

**Published:** 2012-08-15

**Authors:** Elizabeth M Poole, Shelley S Tworoger, Susan E Hankinson, Heather J Baer

**Affiliations:** 1 Department of Medicine, Channing Laboratory, Brigham and Women's Hospital and Harvard Medical School, 181 Longwood Ave, Boston, MA 02115, USA; 2 Department of Epidemiology, Harvard School of Public Health, Boston, MA 02115, USA; 3 Division of General Medicine and Primary Care, Brigham and Women’s Hospital, Boston, MA 02115, USA; 4 Department of Medicine, Harvard Medical School, Boston, MA 02115, USA; 5 Department of Public Health, University of Massachusetts Amherst, Amherst, MA 02115, USA

**Keywords:** IGF-1, IGFBP-1, IGFBP-3, Birthweight, Body size, Adolescence

## Abstract

**Background:**

Early life body size and circulating levels of IGF-1 and IGFBP-3 have been linked to increased risks of breast and other cancers, but it is unclear whether these exposures act through a common mechanism. Previous studies have examined the role of *IGF-1* and *IGFBP-3* genetic variation in relation to adult height and body size, but few studies have examined associations with birthweight and childhood size.

**Methods:**

We examined whether htSNPs in *IGF-1* and the *IGFBP-1*/*IGFBP-3* gene region are associated with the self-reported outcomes of birthweight, body fatness at ages 5 and 10, and body mass index (BMI) at age 18 among healthy women from the Nurses’ Health Study (NHS) and NHSII. We used ordinal logistic regression to model odds ratios (ORs) and 95% confidence intervals (CI) of a one category increase for birthweight and somatotypes at ages 5 and 10. We used linear regression to model associations with BMI at age 18.

**Results:**

Among 4567 healthy women in NHS and NHSII, we observed no association between common *IGF-1* or *IGFBP-1*/*IGFBP-3* SNPs and birthweight, body fatness at ages 5 and 10, or BMI at age 18.

**Conclusions:**

Common *IGF-1* and *IGFBP-1*/*IGFBP-3* SNPs are not associated with body size in early life.

## Background

Although early life body size and circulating IGF-1 and IGFBP-3 have been linked to increased risks of breast cancer [1-3], it is unclear whether these exposures act through a common mechanism. We recently reported that increased birthweight and decreased body size in youth and adolescence were associated with higher IGF-1 and IGFBP-3 levels in adulthood [4], suggesting that early life body size may act on breast cancer risk through its influence on hormone levels in adulthood. Further, although genetic variability in *IGF-1* and *IGFBP-3* has been linked to their circulating levels [5-7], it is unclear whether this same variation may influence childhood body size. Some previous studies of *IGF-1* and *IGFBP-3* genetic variation in relation to adult height and body size reported associations with a putative functional microsatellite polymorphism [6,8-10], but few studies have examined birthweight [11-13] or childhood body size [14]. Further, the studies that examined birthweight examined only the microsatellite polymorphism associated with adult height in previous studies [11-13]. In this study, we investigated potential associations of haplotype tagging SNPs in *IGF-1* and the *IGFBP-*1/*IGFBP-3* gene region with birthweight, body fatness at ages 5 and 10, and body mass index (BMI) at age 18, to determine whether genetic variation could explain the observed relations of early life body size with circulating IGF levels in adulthood.

## Methods

### Study populations

The NHS cohort was established in 1976 among 121,700 US female registered nurses, ages 30 to 55 years; NHSII was established in 1989 among 116,430 female registered nurses, ages 25 to 42 years. All women completed an initial questionnaire about their lifestyle factors, health behaviors, and medical history, and have been followed biennially by questionnaire. From 1989 to 1990, 32,826 NHS participants (ages 43 to 70 years) provided blood samples and completed a short questionnaire [15]. Blood was processed and separated into plasma, red blood cell, and white blood cell components. From 2002 to 2004, kits to collect buccal cells were received from 33,040 NHS women (ages 54–84) had not previously provided a blood sample and had completed the 2000 questionnaire. DNA was extracted and purified upon sample receipt.

Between 1996 and 1999, 29,611 NHSII participants (ages 32 to 54 years) provided blood samples and completed a short questionnaire [16]. Briefly, premenopausal women, either provided a luteal blood sample 7 to 9 days before the anticipated start of their next cycle (n = 18,521) or a single 30-mL untimed blood sample (n = 11,090). NHSII samples were processed identically to the NHS samples. All study participants provided informed consent. This study was approved by the Committee on the Use of Human Subjects in Research at the Brigham and Women’s Hospital and the Harvard School of Public Health (Boston, MA).

The current analysis includes women with available DNA who were controls from 7 nested case–control studies of *IGF-1* and *IGBFP-3* SNPs and risk of various chronic diseases, including benign breast disease [17], breast cancer [5], endometrial cancer [18], myeloma [19], and ovarian cancer [20] (N=4567).

### Body size and covariate information

Body size and covariate information was obtained from the questionnaire completed at sample collection and biennial study questionnaires. Birthweight was collected in 1992 (NHS) and 1991 (NHSII). In the NHS, the correlation between the participant’s self-reported birthweight and that reported by her mother was 0.77 [2]. In 1988 (NHS) and 1989 (NHSII), women were asked to choose one of nine diagrams (somatotypes) [21] that best depicted their body fatness at ages 5 and 10, with higher levels indicating larger body size. Among older women (aged 71–76) in another study population, the correlations between recalled somatotype and measured BMI were 0.57 at age 5 and 0.70 at age 10; the correlations were similar after controlling for current BMI [22]. BMI at blood draw and at age 18 (asked in 1980 for NHS and in 1989 for NHS2) were calculated as self-reported weight in kilograms divided by self-reported height (collected at baseline) in meters squared.

We considered a woman to be premenopausal at sample collection if (1) she gave a luteal sample (NHSII only), (2) her periods had not ceased, or (3) she had at least one ovary and was 47 years or younger (nonsmokers) or 45 years or younger (smokers). We considered a woman to be postmenopausal if (1) her natural menstrual periods had ceased permanently, (2) she had a bilateral oophorectomy, or (3) she had at least one ovary and was 56 years or older (nonsmokers) or 54 years or older (smokers). The age cutoffs represent the age when 90% of women with intact ovaries in the cohorts were premenopausal or postmenopausal, respectively. The remaining women, most of whom had a simple hysterectomy and were 48 to 55 years old, were considered to be of unknown menopausal status.

### SNP selection and genotyping

SNP selection and genotyping have been described previously [5,17,19,20]. Briefly, haplotype tagging SNPs (htSNPs) were identified by the Breast and Prostate Cancer Cohort Consortium (BPC3).^a^ In *IGF-1*, 154 SNPs (56 SNPs in *IGFBP-1*/*IGFBP-3*) were genotyped in a panel representing several racial groups. Of these, 64 *IGFBP-1* and 36 *IGFBP-1*/*IGFBP-3* SNPs passed quality control and were confirmed to be SNPs. From these remaining SNPs, the expectation-maximization algorithm was used to select 14 *IGF-1* and 12 *IGFBP-1*/*IGFBP-3* SNPs that were predicted to tag the common haplotypes in Caucasian populations (r_h_^2^ > 0.85). Four additional SNPs in *IGFBP-1*/*IGFBP-3* were included in the BPC3 genotyping, in which the NHS and NHSII nested case–control samples for breast cancer were included.

DNA extraction and genotyping were performed at the Dana–Farber Cancer Institute/Harvard Cancer Center High Throughput Genotyping Core, a unit of the Harvard–Partners Genotyping Facility. DNA was extracted using a QIAamp 96 DNA Blood Kit (Qiagen). Genotyping assays for the 22 SNPs were performed by the 5’ nuclease assay (Taqman) on the Applied Biosystems Prism 7900HT Sequence Detection System (Applied Biosystems, Foster City, CA). Taqman primers, probes and conditions for genotyping assays are available upon request. Laboratory personnel were blinded to case–control status, and duplicate samples (10% of sample size) were inserted to validate genotyping procedures. More than 95% of the samples were successfully genotyped for each polymorphism and there were no discordant quality control sets.

### Statistical analysis

We used ordinal logistic regression models to analyze the association between *IGF-1* and *IGFBP-1/IGFBP-3* SNPs and body size (birthweight, somatotypes at ages 5 and 10, and average somatotype at ages 5 and 10). Birthweight was categorized as <5.5 lbs, 5.5-6.9 lbs, 7–8.4 lbs, 8.5-9.9 lbs, or 10+ lbs, based on the questionnaire categories. As a secondary analysis, we examined the associations with low birth weight (<5.5 lbs) vs. all others using logistic regression. Somatotypes at ages 5 and 10 were categorized as diagram 1, 2, 3, 4, and 5+. To assess the fit of ordinal logistic regression models, we conducted the score test for proportional odds. Since BMI at age 18 was calculated continuously, we used linear regression to assess the associations between *IGF* variability and this outcome. SNPs were included in the model as ordinal terms with values from 0 to 2 (i.e. as one variable coded 0 for the homozygous major allele genotypes, 1 for heterozygous genotypes, and 2 for homozygous variant genotypes). Additionally, we combined SNPs using two methods. First, 11 SNPs (rs1520220, rs35767, rs7965399, rs2195239, rs2946834, rs2854744, rs2854746, rs3110697, rs2270628, rs2960436, rs2132570) associated with circulating IGF-1 or IGFBP-3 levels previously [5,7,23] were combined to make a SNP score. The allele that was associated with higher levels was considered the “risk” allele. Having one copy of the risk allele added one to the score; two copies added two to the score. Second, we used reduced rank regression [24] to construct a score of IGF-1 and IGFBP-3 SNPs that explained variability in measured plasma IGF-1 and IGFBP-3 in the NHS and NHSII participants who had both plasma levels and genotypes. We applied the scores to all women with genotypes and assessed associations with both scores using ordinal logistic regression (for birthweight and somatotypes) or linear regression (for BMI at age 18) as described above.

All models were adjusted for age at blood draw or cheek cell collection, menopausal status at collection and reference date, postmenopausal hormone use at collection and reference date, and DNA source (blood vs. cheek). The two cohorts were analyzed separately and combined using random effects meta-analysis. All p-values were two-sided and considered statistically significant if ≤0.05. Ordinal logistic regression analyses were conducted in STATA 11.0 (STATACorp, College Station, TX). Linear regression analysis and meta-analyses were conducted using SAS version 9.1 (SAS Institute, Cary, NC).

## Results

Among genotyped NHS participants, 12% had a birthweight <5.5 lbs and 3% had a birthweight of 10 lbs or more (Table 1). Mean somatotypes at ages 5 and 10 were 2.3 and 2.5, respectively, and mean BMI at age 18 was 21.2. Among genotyped NHSII participants, 9% had a birthweight <5.5 lbs and 1% had a birthweight of 10 lbs or more. Mean somatotypes at ages 5 and 10 were 2.5 and 2.8, respectively, and mean BMI at age 18 was 21.1. NHS participants were older than NHSII participants at blood draw/buccal cell collection and were more likely to be postmenopausal. 10% of NHS samples were from buccal cell samples; all other participants had a blood sample.

**Table 1:** Age standardized characteristics at blood draw/buccal cell collection and control selection

	**NHSI**	**NHSII**
	**(n = 3499)**	**(n = 1068)**
Age at blood draw/buccal cell collection, mean (SD)^a^	59.6 (7.7)	44.9 (4.3)
Somatotype at age 5^b^, mean (SD)	2.3 (1.3)	2.5 (0.9)
Somatotype at age 10^b^, mean (SD)	2.5 (1.3)	2.8 (1.0)
BMI at age 18, mean (SD)	21.2 (2.7)	21.1 (2.2)
Birthweight^b^, N (%)		
<5.5 lbs	329 (12.0)	84 (8.8)
10+ lbs	79 (2.9)	12 (1.3)
DNA from blood, N (%)	3132 (89.5)	1068 (100)
Menopausal status/Post-menopausal hormone (PMH) use at blood draw/buccal cell collection^b^, N (%)		
Premenopausal	764 (24.0)	680 (68.2)
Postmenopausal-PMH non-user	1574 (49.5)	136 (13.6)
Postmenopausal-PMH user	843 (26.5)	182 (18.2)
Menopausal status/Post-menopausal hormone (PMH) use at reference date^b^, N (%)		
Premenopausal	508 (16.0)	479 (47.7)
Postmenopausal-PMH non-user	1479 (46.8)	37 (3.7)
Postmenopausal-PMH user	1171 (37.1)	487 (48.6)

^a^Value is not age-standardized.

^b^Calculated among those with known values.

NHS:7% were missing information on somatotype at age 5, 6% were missing information on somatotype at age 10, and 22% were missing information on birthweight. 9% were missing menopausal status at blood draw and 9% were missing menopausal status at selection.

NHSII:1% were missing information on somatotype at age 5, 1% were missing information on somatotype at age 10, and 11% were missing information on birthweight. 7% were missing menopausal status at blood draw and 6% were missing menopausal status at selection.

In general, there was no association between genetic variability in *IGF-1* or *IGFBP-1*/*IGFBP-3* and body size at birth or in childhood (Table 2). *IGF-1* rs35767 was associated with decreased BMI at age 18 (p = 0.03) and rs5742665 was associated with a slight increase in BMI at age 18 (p = 0.04). We also observed significant associations with low birthweight (data not shown), however, none of these associations remained significant after Bonferroni correction. Although Table 2 shows the associations for average somatotype at ages 5 and 10, results were similar when somatotypes at ages 5 and 10 were analyzed separately (data not shown). There was no association with either of the SNP scores after adjustment for multiple comparisons (data not shown).

**Table 2:** **Associations between SNPs in IGF-1 and IGFBP-3 and body size in early life**^**a**^

			**Birthweight**			**Average somatotype**			**BMI at age 18**		
						**at ages 5 and 10**					
**Gene**	**SNP**	**MAF**	**OR**	**95% CI**	**p**	**OR**	**95% CI**	**p**	**β**	**SE**	**p**
IGF-1	rs1996656	0.18	0.96	(0.80-1.15)	0.64	1.03	(0.93-1.14)	0.57	−0.0068	0.0050	0.17
	rs4764695	0.50	1.06	(0.97-1.16)	0.16	1.01	(0.93-1.10)	0.75	0.0017	0.0044	0.70
	rs4764876	0.26	0.90	(0.76-1.08)	0.27	1.02	(0.93-1.11)	0.65	0.0045	0.0032	0.16
	rs2946834	0.32	0.93	(0.85-1.02)	0.12	1.00	(0.92-1.08)	0.91	0.0020	0.0029	0.49
	rs1520220	0.18	0.93	(0.83-1.04)	0.18	0.98	(0.87-1.10)	0.72	−0.0037	0.0036	0.30
	rs1549593	0.13	1.02	(0.89-1.16)	0.79	0.95^b^	(0.72-1.26)	0.73	−0.0007	0.0063	0.91
	rs5742665	0.13	0.98	(0.83-1.15)	0.83	1.00	(0.90-1.13)	0.94	0.0103	0.0051	0.04
	rs2373722	0.07	0.91	(0.77-1.08)	0.28	0.88	(0.71-1.08)	0.21	−0.0027	0.0053	0.61
	rs10735380	0.27	0.93	(0.83-1.04)	0.21	0.98	(0.90-1.06)	0.58	−0.0031	0.0032	0.34
	rs2195239	0.23	0.94	(0.83-1.08)	0.39	1.01	(0.92-1.10)	0.85	−0.0043^b^	0.0080	0.59
	rs1019731	0.13	1.06	(0.93-1.21)	0.35	1.09	(0.98-1.23)	0.12	0.0021	0.0041	0.61
	rs12821878	0.22	1.11	(0.99-1.23)	0.06	1.09	(0.94-1.28)	0.26	0.0030	0.0034	0.38
	rs35767	0.16	0.98	(0.87-1.12)	0.81	0.89	(0.77-1.04)	0.14	−0.0080	0.0038	0.03
	rs7965399	0.04	1.01	(0.75-1.38)	0.92	0.90	(0.75-1.09)	0.30	−0.0026	0.0067	0.70
IGFBP-1/IGFBP-3	rs4619	0.35	1.02	(0.91-1.14)	0.75	0.99	(0.84-1.17)	0.93	−0.0018	0.0029	0.54
	rs2201638	0.03	0.92	(0.57-1.48)	0.73	1.05	(0.71-1.56)	0.80	0.0257	0.0156	0.10
	rs1065780	0.38	1.02	(0.93-1.11)	0.74	1.02	(0.94-1.11)	0.55	−0.0002	0.0039	0.95
	rs1553009	0.20	1.04	(0.94-1.17)	0.44	1.10	(1.00-1.21)	0.06	0.0042	0.0035	0.23
	rs35539615	0.23	0.98	(0.87-1.10)	0.71	0.95	(0.85-1.06)	0.34	0.0018	0.0036	0.62
	rs1908751	0.30	0.97	(0.88-1.07)	0.56	0.95	(0.87-1.03)	0.20	0.0061	0.0031	0.05
	rs4988515	0.04	1.13	(0.91-1.40)	0.27	1.08	(0.89-1.33)	0.43	−0.0018	0.0111	0.87
	rs10228265	0.31	0.94	(0.86-1.03)	0.19	0.98	(0.90-1.06)	0.59	−0.0043	0.0030	0.15
	rs2270628	0.19	1.01	(0.84-1.23)	0.89	0.96	(0.87-1.06)	0.38	−0.0052	0.0064	0.42
	rs6670	0.21	0.93	(0.84-1.03)	0.18	0.99	(0.90-1.09)	0.82	0.0033	0.0043	0.44
	rs2453839	0.20	0.97	(0.86-1.08)	0.54	1.02	(0.84-1.25)	0.84	0.0113^b^	0.0086	0.19
	rs3110697	0.42	0.98	(0.90-1.07)	0.63	0.94	(0.82-1.09)	0.43	−0.0055^b^	0.0081	0.50
	rs2854746	0.40	1.09	(1.00-1.20)	0.06	1.02	(0.94-1.10)	0.65	0.0003	0.0029	0.91
	rs2854744	0.47	1.09	(0.90-1.32)	0.39	1.01	(0.94-1.10)	0.73	−0.0029	0.0032	0.37
	rs2132570	0.22	0.96	(0.81-1.15)	0.69	0.92	(0.83-1.03)	0.16	−0.0048	0.0049	0.33
	rs2960436	0.46	1.12	(0.93-1.34)	0.25	0.99	(0.91-1.08)	0.89	−0.0023	0.0046	0.61

^a^Birthweight was categorized as <5.5 lbs, 5.5-6.9 lbs, 7-8.4 lbs, 8.5-9.9 lbs, and 10+ lbs. Somatotypes at age 5 and 10 were categorized as 1,2,3,4,5+. BMI at age 18 was log-transformed and continuous. Birthweight and somatotypes at age 5 and 10 were analyzed in ordinal logistic regression models; BMI at age 18 was analyzed in a linear regression model. All models were adjusted for age at blood draw/cheek collection, menopausal status at blood draw/cheek collection and case selection, post-menopausal hormone use at blood draw/cheek collection and control selection, and DNA source (NHS only).

^b^The NHS and NHSII cohorts were combined using meta-analysis techniques. These estimates had P-het <0.05, indicating heterogeneity between the cohorts.

Abbreviations: SNP: single nucleotide polymorphism; IGF-1: insulin-like growth factor 1; IGFBP-3: insulin-like growth factor binding protein 3; BMI: body mass index; MAF: minor allele frequency; OR: Odds Ratio; CI: confidence interval; SE: standard error.

## Discussion

In this study of 4567 women from the NHS and NHSII cohorts, we observed no association between genetic variability in *IGF-1* and *IGFBP-1*/*IGFBP-3* and birth weight or body fatness in childhood and adolescence. Previous studies of the association between *IGF-1* or *IGFBP-1*/*IGFBP-3* and early life body size were limited to a putative functional microsatellite polymorphism in the *IGF-1* promoter. This polymorphism has been associated with increased weight, BMI, and fat mass in a Dutch cohort of children ages 11 to 13, particularly among female children [14]. Further, this polymorphism was associated with decreased birthweight [11] in one study, but this was not confirmed in two other studies [12,13]. We did not genotype this polymorphism in our study, but instead focused on htSNPs that capture genetic variability across the *IGF-1* and *IGFBP-1*/*IGFBP-3* loci.

Two studies have examined tagSNPs in these genes in relation to adult height in Caucasians [8,9]. The first [8] examined 13 SNPs in *IGF-1* and 1 SNP in *IGFBP-3* in a panel of 2189 individuals above the 90^th^ percentile or below the 10^th^ percentile of height. Any associations observed in this panel were then tested in two additional study populations. No associations were noted this study. The second study [9] used a family-based approach and reported that two SNPs, rs5742694 and rs2033178, were associated with adult height, particularly among females. We did not genotype these SNPs in our study. However, rs2033178 is in perfect LD with rs2373722, which we did assess. We observed no associations between this SNP and any of the body size measures in our study.

An important limitation of this study is that we did not genotype the *IGF-1* promoter microsatellite polymorphism, which has been associated with circulating *IGF-1* levels [6,10]. However, most previous studies have not observed this polymorphism to be associated with childhood body size. Thus, we focused on genetic variability across the *IGF-1* and *IGFBP-1*/*IGFBP-3* loci. Further, our measures of birthweight and childhood size are based on self-report. However, we have shown previously that these measures are highly correlated with actual size [2,22], indicating that they are reliable measures. A major strength of this study is that we had adequate power to detect even modest associations. For example, for birthweight, for which we had the lowest number of study participants (N = 3640), we had over 80% power to detect modest associations (β = 0.0033) for minor allele frequencies as low as 0.04 at α = 0.001 (Bonferroni corrected for multiple comparisons).

## Conclusions

This study comprehensively examined genetic variation in *IGF-1* and *IGFBP-1*/*IGFBP-3* in relation to birthweight and body size in childhood and adolescence in over 4200 women. We observed no associations between *IGF-1* or *IGFBP-1*/*IGFBP-3* SNPs and body size, suggesting that previously observed associations between childhood body size and adult IGF-1 levels likely are not due to underlying genetic variability in IGF-related genes. However, the lack of association should be confirmed in studies which directly measured birthweight and body size at various ages in youth and adolescence.

## Endnote

^a^http://cgf1.nci.nih.gov/cohort.cfm.

## Abbreviations

BMI: Body mass index; CI: Confidence interval; IGF-1: Insulin-like growth factor 1; IGFBP-1: Insulin-like growth factors binding protein 1; IGFBP-3: Insulin-like growth factors binding protein 3; NHS: Nurses’ Health Study; NHSII: Nurses’ Health Study II; OR: Odds ratio; SNP: Single nucleotide polymorphism.

## Competing interests

The authors declare that they have no competing interests.

## Authors’ contributions

EP carried out the data analysis and drafted the manuscript. ST helped design the study, oversaw the data analysis, and helped to write the manuscript. SH contributed to study design and provided feedback on results. HB conceived of the study, obtained funding for the study, and contributed to manuscript preparation. All authors read and approved the final manuscript.

## Pre-publication history

The pre-publication history for this paper can be accessed here:

http://www.biomedcentral.com/1471-2458/12/659/prepub
